# Crosslinked Chitosan Nanoparticles with Muco-Adhesive Potential for Intranasal Delivery Applications

**DOI:** 10.3390/ijms24076590

**Published:** 2023-04-01

**Authors:** Mariacristina Gagliardi, Sara Chiarugi, Chiara De Cesari, Giulia Di Gregorio, Alessandra Diodati, Laura Baroncelli, Marco Cecchini, Ilaria Tonazzini

**Affiliations:** 1 National Enterprise for nanoScience and nanoTechnology (NEST), Nanoscience Institute—National Research Council (CNR) and Scuola Normale Superiore, Piazza San Silvestro 12, 56127 Pisa, Italy; 2 Institute of Neuroscience, National Research Council (CNR), Via Giuseppe Moruzzi 1, 56124 Pisa, Italy; 3 Department of Developmental Neuroscience, Scientific Institute Stella Maris Foundation, Viale del Tirreno 331, Calambrone, 56128 Pisa, Italy

**Keywords:** chitosan nanoparticles, intranasal administration, drug delivery, mucoadhesion

## Abstract

Intranasal drug delivery is convenient and provides a high bioavailability but requires the use of mucoadhesive nanocarriers. Chitosan is a well-established polymer for mucoadhesive applications but can suffer from poor cytocompatibility and stability upon administration. In this work, we present a method to obtain stable and cytocompatible crosslinked chitosan nanoparticles. We used 2,6-pyridinedicarboxylic acid as a biocompatible crosslinker and compared the obtained particles with those prepared by ionotropic gelation using sodium tripolyphosphate. Nanoparticles were tested to evaluate the size and the surface charge, as well as their stability in storage conditions (4 °C), at the nasal cavity temperature (32 °C), and at the body temperature (37 °C). The crosslinked chitosan nanoparticles showed a size around 150 nm and a surface charge of 10.3 mV ± 0.9 mV, both compatible with the intranasal drug administration. Size and surface charge parameters did not significantly vary over time, indicating the good stability of these nanoparticles. We finally tested their cytocompatibility in vitro using SHSY5Y human neuroblastoma and RPMI 2650 human nasal epithelial cells, with positive results. In conclusion, the proposed synthetic system shows an interesting potential as a drug carrier for intranasal delivery.

## 1. Introduction

Over the past few decades, several studies have indicated the nasal mucosa as a promising therapeutic pathway for drug delivery [1]. In addition to non-invasive and painless administration, intranasal drug delivery can provide numerous benefits, such as easy accessibility, bypassing the hepatic and metabolic first-pass effects, low costs, good patient compliance, and the possibility to target the brain [2].

Mucoadhesion is a fundamental aspect of intranasal drug delivery [3]. Mucoadhesion is the ability of a substance to adhere to the mucosa of the nasal cavity [4], and it depends on the chemical, physical, and mechanical properties of the substance [5] and on the interactions with the nasal mucus [6]. A strong mucoadhesion can enhance the residence time of the active principle in the nasal cavity [7], boosting drug absorption and efficacy [8].

The optimization of mucoadhesion-related properties can be successfully exploited for topical drug administration [9] and in brain targeting [10]. The olfactory epithelium and the trigeminal nerve pathways work as a direct route for nose-to-brain delivery [3], enabling the rapid and efficient absorption of active principles and potentially reducing the adverse effects of systemic administration [11].

The maximization of the effective drug dose in brain targeting is fundamental, and the encapsulation of the drug in mucoadhesive vectors, such as polymeric nanoparticles (**NPs**), is a valid strategy for poorly mucoadhesive active principles [12] and non-invasive brain delivery. The recent literature reports some interesting polymer, lipid, and inorganic NP-based applications for nose-to-brain delivery [13].

Several kinds of interactions, such as electrostatic interactions, hydrogen bonding, and van der Waals forces, participate in mucoadhesion [9]. The surface properties of the NPs, in particular the charge and the degree of hydrophilicity, play a crucial role in mucoadhesion [14,15]. Positively charged NPs strongly interact with the negatively charged mucus, leading to improved mucoadhesion [16]. NP size can also affect mucoadhesion [17]: here smaller nanoparticles have a maximized surface/volume ratio, which can increase their interaction with the mucus [18].

Recently, an optimized semi-synthetic nanocarrier, named mucosome and composed of mucin [19], was developed and tested with interesting capabilities in terms of cytocompatibility, drug delivery, and biodistribution.

Mucoadhesive polymers are materials with intrinsic mucoadhesive properties [20]. Fully synthetic polymeric nanocarriers were also tested with significant results [20,21]. Chitosan (**CHI**) is a natural, biocompatible, and biodegradable polymer that is derived from chitin, which is found in the exoskeleton of crustaceans. Chitosan has a positive charge, which allows it to interact with the negatively charged nasal mucus, thereby improving the mucoadhesion of nanoparticles. For such properties, CHI is a widely used mucoadhesive polymer for nasal drug delivery of insulin, vaccines, pain medications, peptides, and drugs for neurological disorders [22]. Several efforts have been recently focused on the development of CHI nanodelivery systems for nose-to-brain applications, such as hydrogels, emulsions, and NPs [7,13,23]. However, cationic polymer/chitosan vectors have failed to translate into the clinic due to significant in vivo toxicity, which is often material-dependent [24]. Interactions between CHI and mucin, the most present protein in the nasal mucus [19], are mainly electrostatic forces between the cationic polymer and the negatively charged glycoproteins of mucin [14]. Additional forces, such as hydrogen bonding, as well as hydrophobic interactions, strengthen the mucoadhesive nature of CHI [25]. Mucoadhesive properties of CHI are related to its deacetylation degree (DD), which affects both the number of free amino groups and the polymer chain conformation [26]. Several chemical modifications of the CHI macromolecule are currently under investigation to further improve mucoadhesion. The CHI derivatization is currently widely investigated to obtain customized physicochemical features [27]. Trimethylated chitosan [28] and thiolated chitosan [29] are among the more extensively utilized and favored mucoadhesive CHI derivatives.

The present paper reports the development of a novel synthetic scheme to obtain chitosan nanoparticles (CHI-NPs) with elevated mucoadhesion potential and improved cytocompatibility. NPs were obtained by two strategies: (1) ionotropic gelation of CHI by using sodium tripolyphosphate (**CHI-TPP-NPs**); (2) crosslinking of CHI with 2,6-pyridinedicarboxylic acid (DA), a non-toxic dicarboxylic acid that can form ester bonds with the free amines of chitosan (**CHI-DA-NPs**). CHI-NPs were investigated at physicochemical and biological levels.

## 2. Results

### 2.1. Physicochemical Characterization

**Figure 1:**
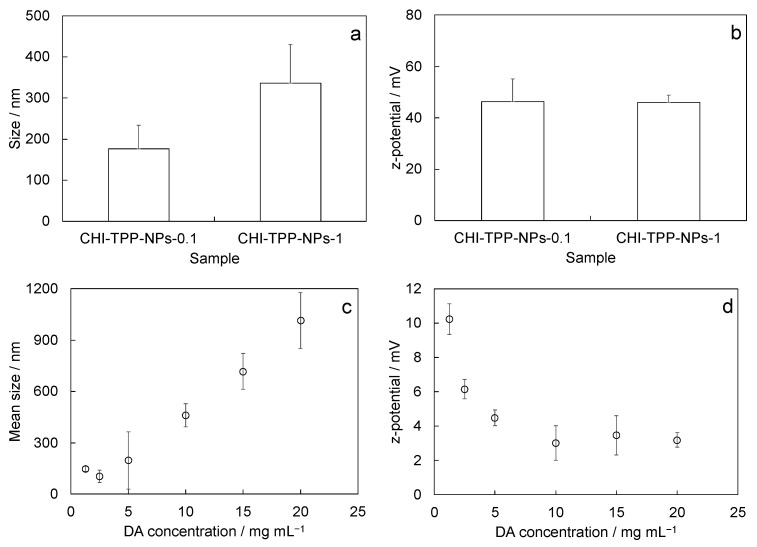
Size and surface charge of produced nanoparticles: (**a**) mean diameter (nm) and (**b**) z-potential (mV) of CHI-TPP-NPs with varying concentrations of TPP solution used for ionotropic gelation (0.1 and 1.0 mg/mL); (**c**) mean size (nm) and (**d**) z-potential (mV) of CHI-DA-NPs synthesized with different DA concentrations. Values are reported as mean ± SEM, replicate experiments correspond to different syntheses, *n* = 6 for CHI-TPP-NPs-0.1, *n* = 7 for CHI-TPP-NPs-1, *n* = 7 for CHI-DA-NPs-1.25.

CHI-NPs obtained by ionotropic gelation with sodium tripolyphosphate (TPP) (CHI-TPP-NPs) were prepared. CHI-TPP-NP size slightly depended on the concentration of the TPP solution used for the preparation, with the size increasing with an increase in the amount of TPP used. The final size values obtained were 177 nm ± 57 nm and 336 nm ± 94 nm for CHI-TPP-NPs-0.1 and CHI-TPP-NPs-1, respectively (Figure 1a). TPP concentration had no significant effects on the surface charge. Obtained results were 46.3 mV ± 8.8 mV and 46.0 mV ± 2.8 mV for CHI-TPP-NPs-0.1 and CHI-TPP-NPs-1, respectively (Figure 1b).

We also developed chitosan crosslinked nanoparticles with 2,6-pyridinedicarboxylic acid (DA) (CHI-DA-NPs). The size of CHI-DA-NPs ranged from 105 nm ± 36 nm to 1015 nm ± 164 nm, with a dependence on the DA concentration (from 1.25 to 20 mg/mL) used for the synthesis (Figure 1c). At the same time, the z-potential of CHI-DA-NPs decreased as the DA concentration increased, from 10.3 mV ± 0.9 mV to 3.2 mV ± 0.4 mV (Figure 1d). After this preliminary screening, we selected the formulation obtained with the lower DA concentration (i.e., 1.25 mg/mL). The choice was due to the dimension (147 nm ± 14 nm) and the positive z-potential (10.3 mV ± 0.9 mV), both of which were found to be the most suitable values for a potential in vivo application (e.g., as nanocarrier for the intranasal delivery of active principles). Further tests were performed on the selected formulation, hereafter named CHI-DA-NPs-1.25.

**Figure 2:**
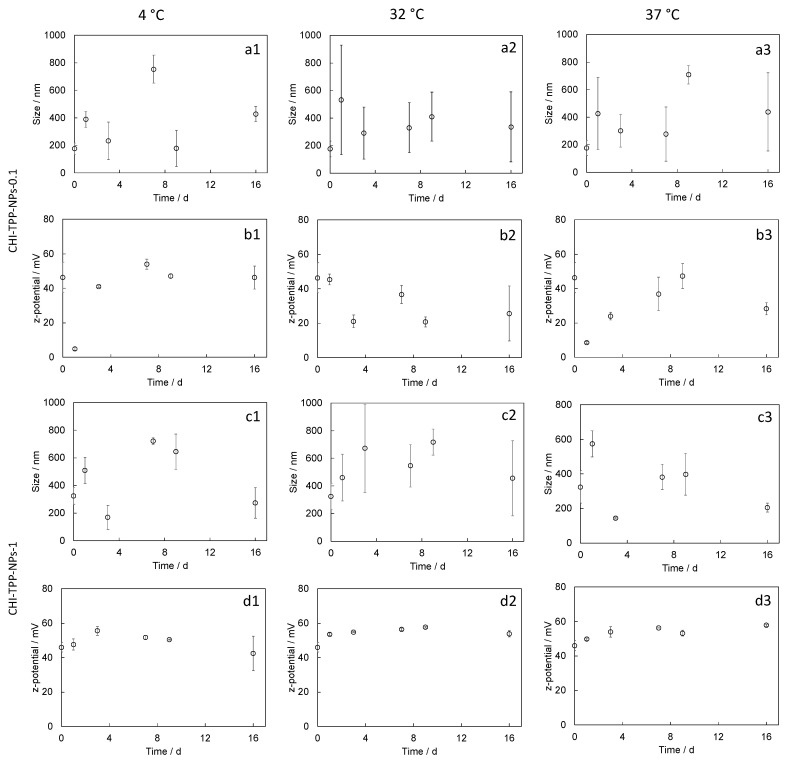
Evaluation of size and z-potential trends measured during stability tests at different temperatures (4 °C, 32 °C, and 37 °C) up to 16 days: (**a1**–**a3**) size and (**b1**–**b3**) z-potential measured for CHI-TPP-NPs-0.1; (**c1**–**c3**) size and (**d1**–**d3**) z-potential measured for CHI-TPP-NPs-1. Values are reported as mean ± SEM.

**Figure 3:**
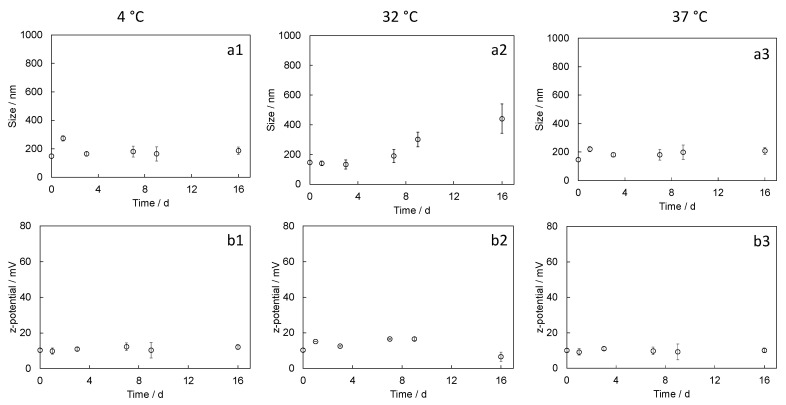
Evaluation of size and z-potential trends measured during stability tests at different temperatures (4 °C, 32 °C, and 37 °C) up to 16 days: (**a1**–**a3**) size and (**b1**–**b3**) z-potential measured for CHI-DA-NPs-1.25. Values are reported as mean ± SEM.

Stability tests were performed to evaluate changes in size or surface charge over time in storage conditions (4 °C), in working conditions at the nasal cavity temperature (32 °C), or in physiological in vivo conditions (37 °C). The size of CHI-TPP NPs, in all the considered temperature conditions, was highly variable over time (Figure 2a,c), with significant fluctuations in the measurements. Values of z-potentials were also highly variable for the system CHI-TPP-NPs-0.1, while the same parameter was less variable in CHI-TPP-NPs-1 (Figure 2b,d). The system CHI-DA-NPs-1.25 showed higher stability, both in terms of size and z-potential, at all the considered temperatures. We have registered only a slight size increase at 32 °C (Figure 3).

### 2.2. In Vitro Cell Tests

Before starting the in vitro characterization, we evaluated how the size of our NPs varied in the presence of the cell culture medium. We used DLS to monitor the size upon the resuspension (0 h) and after 24 h of incubation in cell culture medium supplemented with FBS (10%) at 37 °C (Table 1). The size fluctuated in both the CHI-TPP-NP systems, likely due to aggregation/disaggregation phenomena. The size of CHI-AD-NPs-1.25 was almost stable, with just a slight increasing trend over time, probably due to the interaction with proteins that form a coat over the NP surface.

We then investigated the safety profile of our formulations by measuring the NPs’ effect on cell proliferation and viability in two relevant cell models for eventual intranasal brain delivery, the human neuroblastoma SH cells and the human nasal epithelial RPMI 2650 cells.

We treated cells with different concentrations of CHI-TPP-NPs (2.5, 1, 0.5, 0.25 mg/mL) for 24 h. For the higher concentration treatments (2.5 and 1 mg/mL), some color changes in the culture medium (+phenol red) were observed during the preparation of the solutions, which suggested a slight modification of the pH. We quantified the pH of the NP solutions in the cell medium (Table 2). For both CHI-TPP-NP solutions, the pH varied slightly, and the pH was below pH 7 for the higher concentrations (≥2 mg/mL) (Table 2).

**Figure 4:**
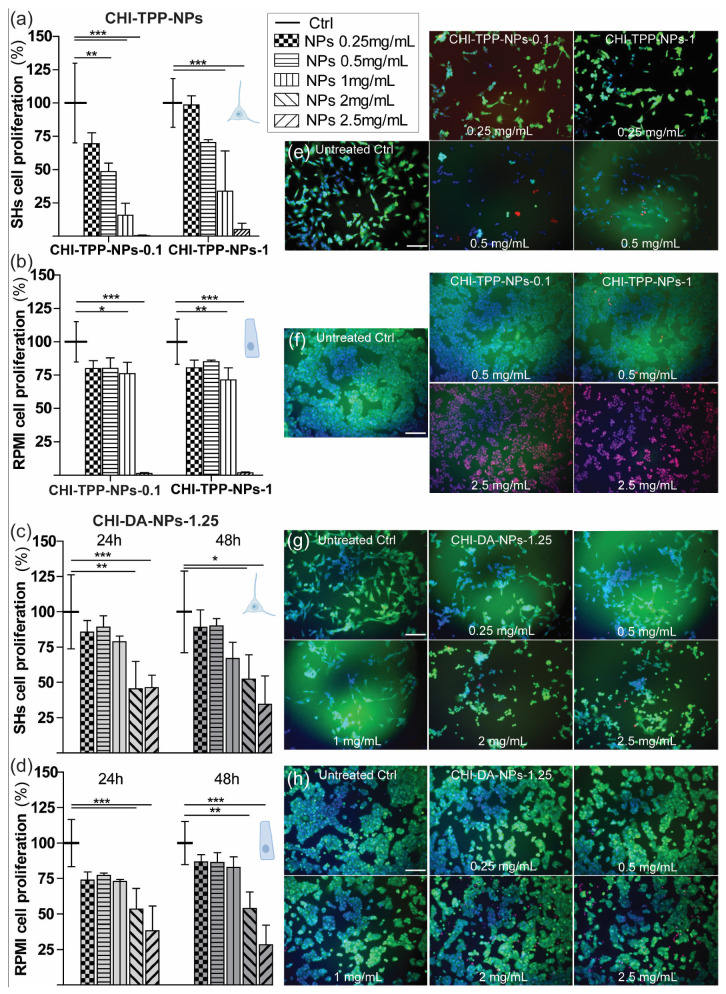
Biocompatibility tests on neuronal and nasal epithelial cells. (**a**,**b**) The proliferation rate of SH cells (**a**) or RPMI-2650 cells (**b**) treated for 24 h with different concentrations (0.25–2.5 mg/mL) of CHI-TPP-NPs formulated with 0.1 mg/mL TPP (left) or 1 mg/mL TPP (right), reported in % over their untreated conditions (Ctrl). */**/*** *p* < 0.05/0.01/0.001, one-way ANOVA, Dunnett’s test. Inset: legend of NP concentrations. (**c**,**d**) The proliferation rate of SH (**c**) or RPMI-2650 (**d**) cells treated with CHI-DA-NPs-1.25 at different concentrations (0.25–2.5 mg/mL) for 24 and 48 h, reported in % over untreated conditions (Ctrl). Data = mean ± SEM, *n* ≥ 3; only Ctrl is reported as mean ± SD, to show the intra-assay variability. (**e**–**h**) Vitality test: adherent vital cells are visible in green (calcein-positive), all cell nuclei in blue, and the level of necrotic/dying cells in red (PI-positive). (**e**,**f**) Fluorescence microscopy images of SHs (**e**) or RPMI-2650 (**f**) at t = 24 h after selected CHI-TPP-NP treatments. (**g**,**h**) Fluorescence microscopy images of SHs (**g**) or RPMI-2650 (**h**) at t = 24 h after all CHI-DA-NP treatments. Scale bar = 100 µm.

Overall, CHI-TPP-NPs-0.1 negatively impacted SH cell proliferation and viability at concentrations within 0.5 mg/mL (*p* < 0.01–0.001 vs. control, Dunnett’s test; Figure 4a left). However, the increase in TPP concentration in the NP formulation slightly improved the NPs’ cell biocompatibility, and CHI-TPP-NPs-1 had no negative effects on neuronal cell proliferation at lower concentrations (≤0.5 mg/mL) (Figure 4a right). In parallel, we also tested CHI-TPP-NPs on RPMI nasal epithelial cells, with overall better results in terms of biocompatibility with respect to those for SHs. CHI-TPP-NPs-0.1 and 1 negatively impacted RMPI-2650 cell proliferation only at 2.5 mg/mL (*p* < 0.001 vs. control, Dunnett’s test; Figure 4b) and slightly at 1 mg/mL (*p* < 0.05/0.01 vs. control, Dunnett’s test; Figure 4b) but did not at lower concentrations.

We then tested CHI-DA-NPs-1.25 at different concentrations (2.5, 2, 1, 0.5, 0.25 mg/mL) for 24 and 48 h, again on SHs and RPMI-2650 (Figure 4c,d). First, CHI-DA-NPs-1.25 showed minor effects on medium pH. For CHI-DA-NPs-1.25 solutions, the pH was almost stable across the initial medium pH (Table 2), being between pH 7.2 ± 0.1 for 2.5 mg/mL and pH 8.1 ± 0.2 for 0.25 mg/mL solution. CHI-DA-NPs-1.25 showed no effects on the proliferation behavior of SHs except for the higher concentrations (2 and 2.5 mg/mL; *p* < 0.01/0.001 vs. control, Dunnett’s test; Figure 4c), with results comparable to the untreated control for all the other concentrations tested up to 48 h (Figure 4c). CHI-DA-NPs-1.25 had similar effects on RPMI-2650 cell proliferation, allowing a standard proliferation within concentrations < 2 mg/mL (Figure 4d), and overall a > 50% proliferation (vs. control condition) for the 2 mg/mL dose.

Finally, we qualitatively checked the vitality status of cells treated with CHI-TPP-NPs and CHI-DA-NPs-1.25 (Figure 4e–h). CHI-TPP-NPs at higher doses were particularly toxic for SHs, with no viable cells visible at the higher doses. Only SHs treated with CHI-TPP-NPs-0.1 and 1 at 0.25 mg/mL (Figure 4e) were well spread and vital (calcein+ green cells), with negligible levels of necrotic cells (PI+ red cells), over the total cells (Hoechst+ blue nuclei). Instead, SHs treated with CHI-DA-NPs-1.25 (at 0.25–2.5 mg/mL) were mainly all viable (Figure 4g). SHs exposed to higher doses of CHI-DA-NPs-1.25 (2 and 2.5 mg/mL) only showed lower cell spreading (i.e., cells were mainly round-shaped) with the presence of some necrotic/apoptotic cells. NPs had similar effects on RPMI nasal cells. CHI-TPP-NPs at the highest dose (2.5 mg/mL) induced complete cell death and at lower doses were better tolerated by these cells (Figure 4f). RPMI cells treated with CHI-DA-NPs-1.25 (at 0.25–2.5 mg/mL) were vital and maintained their normal morphology (Figure 4h): here only cells exposed to the higher doses (2–2.5 mg/mL) showed an increasing level of dying cells (PI+ red cells).

Overall, CHI-TPP-NPs are not highly biocompatible in vitro with neuronal cells, while they show a moderate improvement in their viability profile with nasal cells, at concentrations below 1 mg/mL. The formulation with 1 mg/mL TPP improves the NP safety profile with SH neural cells, but not with RPMI-2650 nasal cells. Overall, CHI-TPP-NPs show significant cell toxicity effects (in particular on SHs), except for the lower concentration (0.25 mg/mL). On the other hand, CHI-DA-NPs-1.25 show an improved safety profile. CHI-DA-NPs-1.25 slightly reduce both neuronal and nasal cells’ proliferation at the higher doses (≥2 mg/mL) but with low effects on cell vitality: in fact, almost all neuronal and epithelial cells treated with all CHI-DA-NPs-1.25 treatments are vital, with normal morphological features.

## 3. Discussion

We developed a novel synthetic scheme to obtain CHI-NPs with improved cytocompatibility and mucoadhesion potential. CHI-NPs crosslinked by DA, a non-toxic dicarboxylic acid, showed optimal characteristics at physicochemical and safety levels: results indicated a better stability and a higher cytocompatibility with neuronal and nasal epithelial cells with respect to those obtained by ionotropic gelation with TPP.

CHI-TPP-NPs were selected as a control because such a system is already known in the literature and because the ionotropic gelation of CHI is well established. We have detected an effect of TPP concentration on the formation of CHI-NPs by ionotropic gelation. Ionotropic gelation involves the interaction between ionic charges on the polymer macromolecules and free ions added to the solution used for nanoparticle preparation. In our case, we have free cationic amines on the CHI molecules and five anionic negative charges for each TPP molecule. The smaller diameter of CHI-TPP-NPs-0.1 with respect to that measured for CHI-TPP-NPs-1 can be attributed to the lower number of CHI macromolecules involved in the formation of a single nanoparticle, due to the lower number of anionic groups. A lower number of TPP molecules reduces the possibility to obtain high crosslinking degrees, and thus CHI macromolecules have high mobility and can form a large number of smaller particles. On the other hand, the surface charge of the obtained nanoparticles did not vary with the TPP concentration. From this result, we can conclude that the amount of TPP used in the preparation of the formulation CHI-TPP-NPs-0.1 is not sufficient to form stable nanoparticles for our aim. A further improvement was obtained by changing the chemical scheme for the formation of the CHI network. We have selected a small molecule to be used as a crosslinker, DA, and we have proposed a crosslinking reaction based on the EDCl/NHS chemistry.

The same trends for size and z-potential were detected for CHI-DA-NPs. However, in the case of CHI-DA-NPs-1.25, measured values of z-potential (10.3 mV ± 0.9 mV) were significantly different from those measured for CHI-TPP-NPs (46.3 mV ± 8.8 mV and 46.0 mV ± 2.8 mV for CHI-TPP-NPs-0.1 and CHI-TPP-NPs-1, respectively). We can conclude that, in the concentration ranges considered in this work, crosslinking sites obtained with DA are significantly more abundant than those obtained in ionotropic gelation with TPP. However, high positive values of z-potential are generally related to poor cytocompatibility [30]; thus, a chemical strategy to reduce the cationic charge of surface NPs can improve this aspect.

Stability tests performed on CHI-TPP-NPs indicated that such particles were not stable. Samples showed very high fluctuations in size at all the temperatures tested.

On the contrary, CHI-DA-NPs-1.25 confirmed the high stability of the prepared nanoparticles, indicating that both the size and the z-potential did not change over time. The stability of the system at 4 °C ensures that nanoparticles can be safely stored in a mild condition after their preparation without any loss of physical properties. Moreover, the stability at 32 °C and at 37 °C at early times indicates that the system could control the potential release of the cargo in vivo, avoiding drug leakage or massive release in correspondence with their disaggregation. The high stability of the system CHI-DA-NPs-1.25 is due to the covalent crosslinking, which is stable in an aqueous environment. Longer test times are needed to define the shelf life of the system in storage conditions and the degradation kinetics of the polymer after the chemical crosslinking.

Here, we chose SH human neuroblastoma cells and RPMI-2650 human nasal epithelial cells to test our NPs in vitro in relevant cell models, in view of their future application as vectors for intranasal administration to the brain. SHs are in fact a valuable model for studying neuronal cell behavior [31,32,33,34], while RPMI-2650 cells resemble the nasal mucosa [35,36], and both these cell types are the cells primarily involved in the nose-to-brain delivery process [3].

CHI-TPP-NPs-0.1 highly impair SH cell proliferation, while CHI-TPP-NPs-1 behave similarly but to a lower extent. Such low biocompatibility with neuronal cells for CHI-TPP-NPs can be due to various factors, such as an excess of positive charge given by the free amines of chitosan, which can make cell membranes unstable [31], or the effects of pH variation of the culture medium below pH 7 [37]. Physiological pH is generally considered to be in the range of 7.2 to 7.4 for normal tissues and pH 7.2–7.4 is ideal for mammalian cells [38], even if these values can vary between cell types and during cultivation, with a larger range suitable for cell viability [37]. In general, cell growth, and particularly SH growth, declines gradually on the acid side of the optimal pH range (e.g., pH 6) [31,37]. In line with this, the increase in the TPP crosslinking agent improved the CHI-TPP-NPs-1 cell biocompatibility in vitro, likely by smoothing the pH variation in treatment solutions and by reducing NPs’ external charges. In fact, CHI-TPP-NPs-1 at lower concentrations (0.25–0.5 mg/mL) do not create significant cell toxicity and allow almost standard cell proliferation. CHI-TPP-NPs had minor effects on RPMI-2650 cell proliferation, if we exclude the 2.5 mg/mL dose, thus suggesting that these cells can better react to pH variations and maybe also to exposure to charges. In line with this, in physiological conditions, the human nose has a pH ≈ 5.6–6.5 [39].

On the other hand, CHI-DA-NPs-1.25 show an optimal safety profile in vitro. CHI-DA-NPs-1.25 slightly reduce both neuronal and nasal cell proliferation only at the higher doses (≥2 mg/mL) but with low effects on cell vitality. RPMI-2650 cells in particular showed standard spreading and morphological features also exposed to the higher doses of CHI-DA-NPs-1.25. Overall, CHI-DA-NPs-1.25 have good physicochemical and in vitro biological features for the proposed application as an intranasal delivery system: a dimensional range around 150 nm and a mild positive z-potential with mucoadhesive potential. In fact, small NPs (<200 nm) need to be uploaded inside cells, and biological barriers [40], as well as positively charged NPs (given by the free amines of chitosan), are important for the interaction with the negatively charged glycoprotein mucins and for mucoadhesion in intranasal delivery [41,42]. The free charges also exert biological toxic effects; in fact, cationic polymer/chitosan nanoparticles have often failed to translate into the clinic due to significant in vivo toxicity [24].

We developed these NPs envisioning repetitive intranasal delivery applications. We therefore purposely avoided the presence of any toxic or potentially toxic chemical in the NP formulation from the beginning: we searched for an alternative crosslinking agent in the place of the well-established glutaraldehyde, which is unfortunately highly toxic for humans. We here exploited DA, a biologically derived dicarboxylic acid: DA is highly present in nature, produced as a secondary metabolite in the core of certain bacterial spores, is readily biodegradable (can be degraded of the simple pyridines) [43,44], and is non-toxic [45]. DA has been recently used in the production of ligands for pharmaceuticals, and it can be recombinantly produced in *E. coli* [45]. Importantly, our CHI-DA-NPs are developed to be maximally safe and yield no hazardous substances via a sustainable synthesis process.

## 4. Materials and Methods

### 4.1. Materials

Medium-molecular-weight chitosan (CHI, deacetylation grade 75–85%; Sigma Aldrich, St. Louis, MO, USA) and sodium tripolyphosphate (TPP, Sigma Aldrich, technical grade) were used in the preparation of CHI-TPP-NPs; CHI, 2,6-pyridinedicarboxylic acid (DA, Sigma Aldrich, purity degree > 99%), N-(3-Dimethylaminopropyl)-N′-ethylcarbodiimide hydrochloride (EDCl, Sigma-Aldrich, commercial grade), and N-Hydroxysuccinimide (NHS, Sigma-Aldrich, purity degree 98%) were used for the synthesis of CHI-DA-NPs. Distilled water (DI), ethanol (Sigma Aldrich, purity > 98%), acetic acid (Sigma Aldrich), and phosphate buffer (PBS, Sigma Aldrich) were used as solvents.

### 4.2. Nanoparticle Preparation via Ionotropic Gelation (CHI-TPP-NPs)

First, 8 mL of DI and 2 mL of TPP solution in water (0.1 mg/mL and 1.0 mg/mL) were added to a glass beaker and mildly stirred. Then, 1 mL of chitosan solution (10 mg/mL, previously extruded with a 0.2 μm syringe filter) was added dropwise. The mixture was stirred for 1 h, and then it was concentrated to 2 mL by using a rotary evaporator under a vacuum. The final nanoparticle dispersion was then dialyzed over PBS for 48 h to eliminate acetic acid and residual TPP (membrane cut-off 14 kDa). The final nanoparticle suspension was stored in a glass vial at 4 °C before use.

### 4.3. Crosslinked Nanoparticle Synthesis with 2,6-Pyridinedicarboxylic Acid (CHI-DA-NPs)

First, 10 mL of DA solution in water (DA concentrations ranged from 1.25 mg/mL to 20.0 mg/mL) was added to a single-neck round-bottom glass reactor and stirred. Then, EDCl and NHS were added to the reactor (0.02 mmol/mL each). The mixture was stirred for 10 min, and then a CHI solution (10 mg/mL in acetic acid 1% *v*/*v*) was added to the reactor. The reaction was maintained under stirring for 30 min, and then the product was dialyzed for 48 h over PBS to restore the pH to a physiological value and to eliminate the residual EDCl, NHS, and DA. The final nanoparticle dispersion was stored in a glass vial at 4 °C before use.

### 4.4. Physicochemical Characterization

#### 4.4.1. Size and Surface Charge

The size of prepared NPs was measured by dynamic light scattering (DLS, Malvern Panalytica Zetasizer, Malvern, UK). A 10 μL sample was diluted in 400 μL of citrate buffer at pH 5 and added to a quartz cuvette for the measure. Surface charge was measured by z-potential. A 10 μL sample was diluted in DI and added to a folded capillary cell for the measure. All the measures were performed in triplicate. Data are reported as average values ± the standard error of the mean (mean ± SEM); at least *n* = 3 independent formulations were analyzed, if not differently stated.

#### 4.4.2. Nanoparticle Stability

Nanoparticle stability was checked at 4 °C (storage condition), 32 °C (average temperature of the nasal cavity [46]), and 37 °C (physiological body temperature) up to 16 days [47]. For stability tests, 50 μL of nanoparticle dispersion with a concentration of 5 mg/mL was added to a plastic Eppendorf tube and maintained at the selected temperature. After fixed times, samples were withdrawn and diluted in 400 μL of PBS (for CHI-DA-NPs-1.25) or water (for CHI-TPP-NPs), and 50 μL of these suspensions was added to a quartz cuvette and measured. After the size measure, the same sample was diluted with 1 mL of DI for z-potential measures. Stability tests were performed in duplicate. We tested *n* = 2 formulations for each type of NPs.

We also monitored the NP size upon resuspension (0 h) and after 24 h of incubation in cell culture medium (DMEM supplemented with FBS 10%) by DLS. NP stock solutions (5 mg/mL) were diluted in medium to a concentration of 2 mg/mL. The prepared suspension was further diluted 1:1 with medium supplemented with FBS 20% to obtain an NP concentration of 1 mg/mL and an FBS concentration of 10% in medium. Then, 60 µL of the solution was added to a quartz cuvette and measured by DLS, immediately or after 24 h at 37 °C. We here tested *n* = 2 formulations for each type of NPs, with similar results.

### 4.5. Functional Characterization

#### 4.5.1. In Vitro Biocompatibility Tests on Neuronal Cells

Human neuroblastoma-derived SH-SY5Y (SH) cells (ATCC HTB-11) were cultured in DMEM medium supplemented with 10% FBS, 4 mM glutamine, and 10 U/mL penicillin–10 mg/mL streptomycin, in standard conditions (37 °C, 95% humidity, 5% CO_2_). Human nasal septum carcinoma RPMI 2650 (RPMI-2650) cells (ATCC CCL-30), kindly provided by Prof. F. Sonvico and A. Bianchera (Univ. of Parma, Italy), were cultured in MEM medium supplemented with 10% FBS, 1% nonessential amino acids, 2 mM glutamine, 10 U/mL penicillin–10 mg/mL streptomycin, and 1 mM sodium pyruvate, as in [36]. Cells were seeded on 96-well plates (6000–8000 cells/well for SH; 25,000–30,000 cells/well for RPMI-2650). The day after, cells were treated with different concentrations of NPs (freshly produced, diluted in cell medium). CHI-TPP-NPs were tested at 2.5, 1, 0.5, and 0.25 mg/mL for 24 h, while CHI-DA-NPs were tested at 2.5, 2, 1, 0.5, and 0.25 mg/mL concentrations, for 24 and 48 h. The pH of the different NP solutions in cell media was quantified, in triplicate for each formulation, using a pH80+DHS pH meter (XS Instruments, Carpi (MO), Italy); data are reported as mean ± SEM, at least *n* = 3 formulations were measured for each NP type.

For cell proliferation assays, after treatments, cells were rinsed twice and assayed using the RealTime-Gl MT Cell Viability Assay (G9712, Promega, Fitchburg, WI, USA), following the instructions (protocol for end-point format). The assay is based on the reduction of NanoLuc substrate from metabolically active cells. After incubation, the solution of each well was moved to a new well in a black plate, and the luminescence was read with a GloMax DISCOVER microplate reader (Promega). The results are reported as percentages of cells with respect to the untreated cells (control condition). We tested each condition in duplicate in the assay, for at least *n* = 3 independent experimental replicates.

Cell live/dead vitality measurements were carried out by the simultaneous imaging of live and dead cells, as in [48]. Calcein (C3100, Molecular probes, Eugene, OR, USA) was used as vital staining, propidium iodide (PI) (P4864, Sigma Aldrich) was used as a necrotic marker, and Hoechst33342 (H3570, Thermofisher, Waltham, MA, USA) was used as cell nucleic acid staining. Here, SHs and RPMI-2650 treated with different NP formulations for 24 h were washed and incubated in a culture medium with calcein (5 µM), PI (1 µg/mL), and Hoechst 33342 (10 µg/mL). Samples were then imaged (within 10 min of treatment) using a Nikon Eclipse-*ti* inverted wide-field fluorescence microscope (Nikon, Tokyo, Japan) equipped with a 20× air Nikon objective (NA 0.45, Plan-Fluor), an incubating chamber (Okolab, Naples, Italy), and a CCD ORCA R2 (Hamamatsu, Iwata City, Japan).

#### 4.5.2. Statistics

Cell proliferation data are reported as average values ± the standard error of the mean (mean ± SEM); only Ctrl is reported as mean ± SD, to show the intra-assay variability. Data were statistically analyzed using GraphPad PRISM program, version 5.00 (GraphPad Software, San Diego, CA, USA). One-way ANOVA analysis (Dunnett’s test) was used to compare different conditions. Statistical significance refers to *p* < 0.05.

## 5. Conclusions

We developed CHI-DA-NPs with optimal physicochemical and biological features for future applications as an intranasal delivery system for the brain. These NPs have a dimensional range of around 150 nm, a mild positive z-potential (with mucoadhesive potential), and an optimal biocompatibility profile in vitro with both human neuronal and nasal epithelial cells and are developed via a maximally safe synthesis process, with no yield of hazardous substances.

## Figures and Tables

**Table 1:** Size (mean diameter, nm) of a set of NP formulations exposed to complete culture medium, upon resuspension (0 h) and after 24 or 48 h of incubation at 37 °C; the size of these NPs upon production (measured in citrate buffer) was 159 ± 53, 342 ± 145, and 159 ± 13 nm, respectively. Values are reported as mean ± SD.

	Size/nm		
at	0 h	24 h	48 h
CHI-TPP-NPs-0.1	268 ± 159	499 ± 33	177 ± 92
CHI-TPP-NPs-1	203 ± 74	329 ± 128	494 ± 89
CHI-DA-NPs-1.25	123 ± 9	149 ± 3	169 ± 4

**Table 2:** pH values of CHI-TPP-NPs and CHI-DA-NPs solutions in cell medium; mean ± SEM, *n* = 3 formulations. NP solutions were prepared in DMEM or MEM medium (supplemented as in Section 4): the initial media pH was 7.9 ± 0.1 for both of them.

	pH (2.5 mg/mL)	pH (2 mg/mL)	pH (1 mg/mL)	pH (0.5 mg/mL)	pH (0.25 mg/mL)
CHI-TPP-NPs-0.1	6.2 ± 0.3	6.6 ± 0.1	7.4 ± 0.3	7.8 ± 0.2	7.9 ± 0.2
CHI-TPP-NPs-1	6.1 ± 0.1	6.5 ± 0.2	7.4 ± 0.2	7.8 ± 0.2	7.8 ± 0.1
CHI-DA-NPs-1.25	7.2 ± 0.1	7.3 ± 0.1	7.7 ± 0.1	7.9 ± 0.1	8.1 ± 0.1

## Data Availability

Data will be publicly published upon the acceptance of the manuscript.
